# Familial Mediterranean fever-related miR-197-3p targets *IL1R1* gene and modulates inflammation in monocytes and synovial fibroblasts

**DOI:** 10.1038/s41598-020-80097-4

**Published:** 2021-01-12

**Authors:** Yeliz Z. Akkaya-Ulum, Tayfun Hilmi Akbaba, Zeynep Tavukcuoglu, Jae Jin Chae, Engin Yilmaz, Seza Ozen, Banu Balci-Peynircioglu

**Affiliations:** 1 Department of Medical Biology, Hacettepe University, Faculty of Medicine, Ankara, Turkey; 2 Inflammatory Disease Section, Metabolic, Cardiovascular, and Inflammatory Disease Genomics Branch, National Human Genome Research Institute, US National Institutes of Health, Bethesda, USA; 3 Division of Rheumatology, Department of Pediatrics, Hacettepe University, Faculty of Medicine, Ankara, Turkey

**Keywords:** Gene silencing, Inflammation

## Abstract

Familial Mediterranean fever (FMF); is an autosomal recessively inherited autoinflammatory disease caused by the mutations in the Mediterranean Fever (*MEFV*) gene. Recent studies have shown that epigenetic control mechanisms, particularly non-coding RNAs, may play a role in the pathogenesis of autoinflammation. microRNAs (miRNAs) are small non-coding RNAs that play critical roles in regulating host gene expression at the post-transcriptional level. The phenotypic heterogeneity of FMF disease suggests that FMF may not be a monogenic disease, suggesting that epigenetic factors may affect phenotypic presentation. Here we examined the potential anti-inflammatory effect of miR-197-3p, which is a differentially expressed miRNA in FMF patients, by using inflammation related functional assays. We monitored gene expression levels of important cytokines, as well as performed functional studies on IL-1β secretion, caspase-1 activation, apoptosis assay, and cell migration assay. These experiments were used to evaluate the different stages of inflammation following pre-miR-197 transfection. Anti-miR-197 transfections were performed to test the opposite effect. 3′UTR luciferase activity assay was used for target gene studies. Our results obtained by inflammation-related functional assays demonstrated an anti-inflammatory effect of miR-197-3p in different cell types (synovial fibroblasts, monocytes, macrophages). 3′UTR luciferase activity assay showed that miR-197-3p directly binds to the interleukin-1beta (IL-1β) receptor, type I (*IL1R1*) gene, which is one of the key molecules of the inflammatory pathways. This study may contribute to understand the role of miR-197-3p in autoinflammation process. Defining the critical miRNAs may guide the medical community in a more personalized medicine in autoinflammatory diseases.

## Introduction

Familial Mediterranean fever (FMF) is an autoinflammatory disease with autosomal recessive inheritance, resulting from mutations in the Mediterranean Fever (*MEFV*) gene found on chromosome 16^1,2^. The *MEFV* gene encodes the pyrin protein^1^. FMF is a disease characterized by serosal, synovial, and cutaneous inflammation as well as recurrent fevers. The most important complication of the disease is secondary amyloidosis^3,4^.

Pyrin is primarily expressed in monocytes and neutrophils, and to a lesser degree in dendritic cells, skin, and synovial fibroblasts. Pyrin consists of 781 amino acids, has a molecular weight of 86 kDa, and is rich in arginine and lysine amino acids^5^. Most of the manifestations currently attributed to FMF are attributed to altered monocyte and neutrophil function. The major causal action of the disease is the pyrin inflammasome activation. This results in high IL-1β production and initiates down-stream pathways to respond to inflammation. This is the key mechanism for inflammation in cells in FMF disease.

Three different phenotypes have been described with FMF^6^. When genotype–phenotype correlation is tested in patients with FMF, large variations can be observed even among patients carrying identical mutations. Phenotypic heterogeneity has been observed among the members of the same family with the same mutation^7^. For example, 15–18% of patients with clinical diagnosis of FMF do not have identified pathogenic variant. Furthermore the “variants of uncertain significance” have different clinical effects in patients^8^. Individuals who have the same genotype, but different phenotypes, may have modifying genes, epigenetic factors, or the environment which could influence the genotype–phenotype relationship seen in FMF.

DNA methylation, histone modifications, and noncoding RNAs (ncRNAs) can be considered as main epigenetic control mechanisms. ncRNAs are classified as small or long, according to the number of nucleotides present in the RNA molecule. microRNAs (miRNAs) are a well-known group of small ncRNAs, that may have a role in the phenotypic heterogeneity observed in genetic diseases. miRNAs are typically 20 to 25 nucleotides in length, do not encode proteins and regulate gene expression. About 2656 mature miRNAs (MiRBase, https://www.mirbase.org, release 22) have been identified in humans and are thought to regulate 60% of protein-encoding gene expression^9^. A single miRNA can regulate the expression of dozens of genes by inhibiting protein translation or degrading mRNA^10^.

miRNA expression patterns of each cell type, contribute to the formation of tissue specific properties and functions. miRNAs can alter gene expression within their own genes and act as intercellular communication molecules. Recent studies have shown that miRNAs can be packaged in exosomes or microparticles, then released from cells into surrounding tissue or circulation^11–13^. Because of these unique features (disease specificity, high stability and accessibility), miRNAs are emerging as important clinical biomarkers useful for the diagnosis of specific diseases and for monitoring treatment responses^14^.

In recent years, many studies have determined that miRNAs may be involved in the regulation of inflammatory processes^15^. miRNAs have been identified in the pathways associated with the immune response, such as the development and differentiation of B and T cells, proliferation of monocytes and neutrophils, activation of the antibody and release of inflammatory regulators^16^. Many common miRNAs have been associated with multiple diseases, such as miR-155 and miR-146a in rheumatoid arthritis (RA), multiple sclerosis (MS), systemic lupus erythematosus (SLE), and bacterial infections^15^. miR-21 is a tributing factor to the pathogenesis of many autoimmune diseases (type I diabetes, psoriasis, MS, SLE, Sjogren syndrome (SS)) and may play an important role in the regulation of autoimmune responses^17–20^.

Several studies have identified miRNAs that may be associated with FMF^21^. These studies focus on the levels of miRNAs without the aid of functional data. Wada et al.^22^ showed that the levels of circulating miRNAs changed during FMF attacks in patients within distinct FMF subgroups, due to specific mutations in the *MEFV* gene. Latsoudis et al.^23^ found a significant increase in the expression of miR-4520a following the silencing of the *MEFV* gene in human pre-monocytic cell line (THP-1) cells. Functional assays showed that this miRNA was bound to the gene which has a Ras homologue enriched in brain (*RHEB*) in the brain. The target of *RHEB* was the main activator in the mammalian target of rapamycin (*mTOR*) signaling pathway of rapamycin. Analysis of blood from ten p.M694V homozygous FMF patients showed increased expression of miR-144-3p, miR-21-5p, miR-4454 and miR-451a in FMF patients compared to healthy controls, while the expression of miR-107, let-7d-5p and miR-148b-3p decreased^24^. miRNA analysis performed in 51 patients, as well as 49 healthy controls, to investigate the effect of miRNAs on the pathogenesis of FMF^25^. Koga et al.^26^ investigated serum samples of FMF patients with and without FMF attacks. The expression of miR-204-3p decreased in the sera of FMF patients. miR-204-3p inhibited inflammatory cytokine release via phosphoinositide 3-kinase gamma (PI3Kγ) pathway and could be used as a good biomarker.

In the presented study, we examined the effect of miR-197-3p on synovial fibroblasts, monocytes, macrophages. We identified this miRNA as one of the differentially expressed miRNAs in whole blood samples of FMF patients^27^ and in inflammation in different cell lines. miR-197-3p levels had been reported in different cancers including p53-dependent lung cancer^28^, non-small cell lung cancer^29^, and hepatocellular carcinoma^30^. miR-197-3p decreased in FMF patients therefore our first experiments were performed with pre-miR-197 transfections to increase the expression level of this miRNA. We have analyzed gene expression levels of inflammatory cytokines and performed functional experiments on IL-1β secretion, caspase-1 activation, apoptosis, and cell migration assays to test the different stages of inflammation following pre-miR-197 transfection. We observed a significant decrease in inflammation parameters in miR-197-3p overexpressing cells. To test the opposite effect, anti-miR-197 transfections were done and it was found that inflammatory genes expression levels and IL-1β secretion significantly increased as expected. Next, we performed candidate gene studies with a 3′ UTR luciferase activity assay and demonstrated that miR-197-3p targets *IL1R1*. These data strongly suggest the potential anti-inflammatory effect of miR-197-3p and its possible influence on the autoinflammation.

## Results

### miR-197-3p modulates the gene expression level of *IL-1β, MEFV, IL-18, TNF-α,* and *TGF-β* in SW982, THP-1 cells, and THP-1 derived macrophages

**Figure 1:**
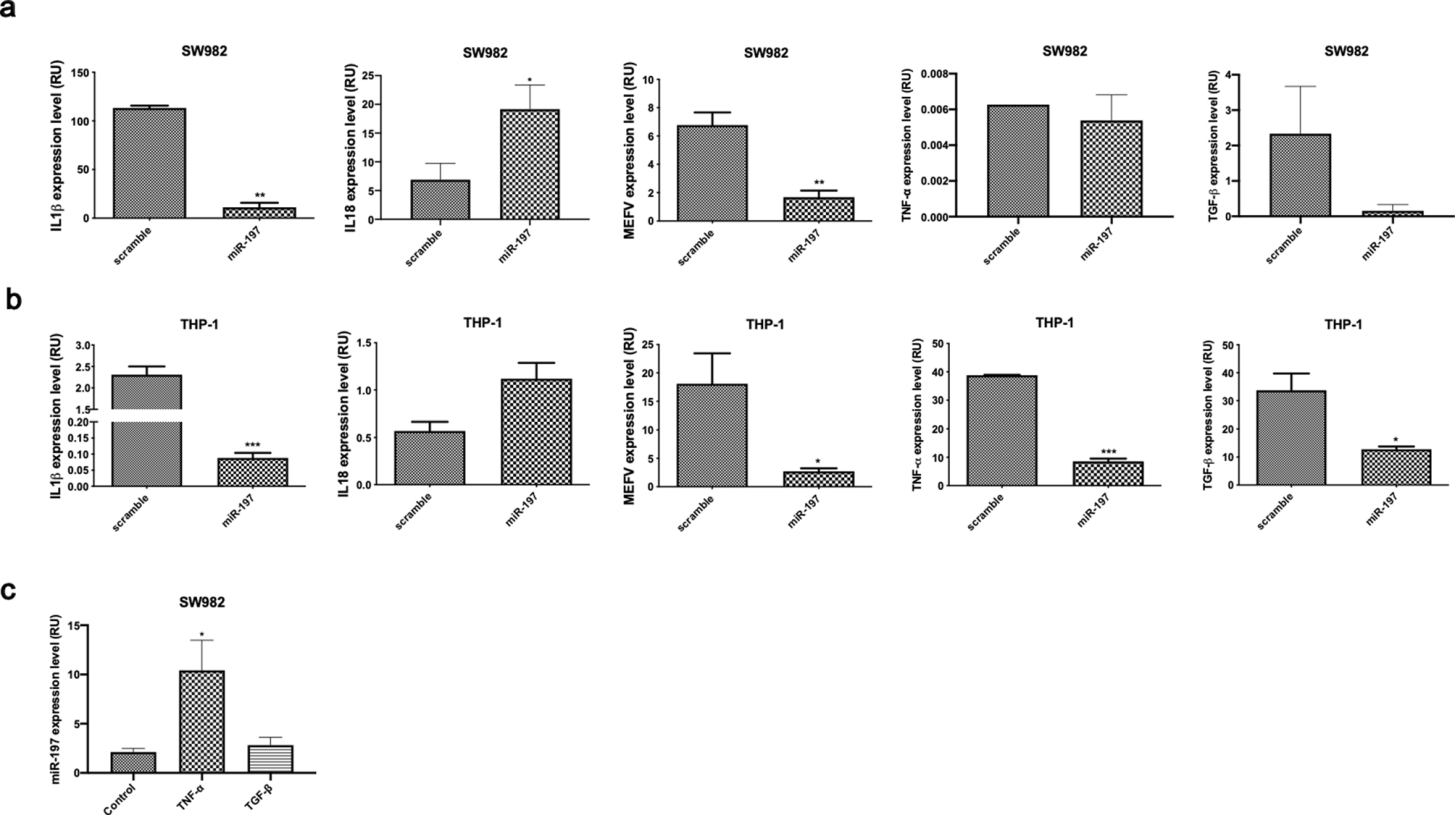
miR-197-3p suppresses the expression of inflammatory cytokines and genes. **(a)** qPCR measurement of *IL-1β*, *IL-18*, *MEFV*, *TNF-α,* and *TGF-β* gene expression levels in miR-197-3p or control sequence-transfected (scramble/mimic control) SW982 cell line. Data represent the mean ± standard deviation from three independent experiments. * *P* < 0.05; ** *P* < 0.01, Student’s t-test (two-tailed). (**b)** qPCR measurement of *IL-1β, IL-18, MEFV, TNF-α,* and *TGF-β* gene expression levels in miR-197-3p or control sequence-transfected (scramble/mimic control) THP-1 cell line. Data represent the mean ± standard deviation from three independent experiments. *P < 0.05; ** *P* < 0.01, Student’s t-test (two-tailed). (**c)** miR-197-3p expression level in SW982 cell line after TNF-α and TGF-β applications. Data represent the mean ± standard deviation from three independent experiments. * *P* < 0.05, Student’s t-test (two-tailed).

In our previous study, miR-197-3p is significantly decreased in homozygous (M694V/M694V) FMF patients compared to controls. This was shown in both miRNA microarray and qRT-PCR analysis^27^. In order to understand the possible anti-inflammatory effect of miR-197-3p, we transfected SW982 cells with pre-miR-197 and checked the expression of inflammatory cytokines and *MEFV* by qPCR. The expression levels of *IL-1β* and *MEFV* genes were decreased in pre-miR-197 transfected cells. *TGF-β* and *TNF-α* expression levels were also non-significantly decreased in pre-miR-197 transfected cells, while *IL-18* expression level was significantly increased (Fig. 1a). We have shown that after transfection of pre-miR-197, the expression level of proinflammatory cytokines and transcription factors decreased significantly by targeting pre-miR-197′s potential target genes to suppress inflammation. Expression analysis supports that this miRNA may be involved in pathways such as cytokine release and stimulation of inflammation through its target gene. Suppressing the target gene suppresses inflammation resulting in an anti-inflammatory effect. In THP-1 cells, the expression levels *of IL-1β, MEFV, TNF-α*, and *TGF-β* genes were decreased in transfected cells while *IL-18* gene expression was increased in a statistically non-significant manner (Fig. 1b). These results were like the findings in SW982. We next investigated the endogenous expression of miR-197-3p following TNF-α and TGF-β stimulation. TNF-α treatment significantly increased the level of miR-197-3p expression, whereas there is no difference in miR-197-3p expression level after TGF-β treatment (Fig. 1c). The increase in miR-197-3p expression following TNF-α stimulation, may be explained with its possible function in suppressing inflammation as an anti-inflammatory molecule.

**Figure 2:**
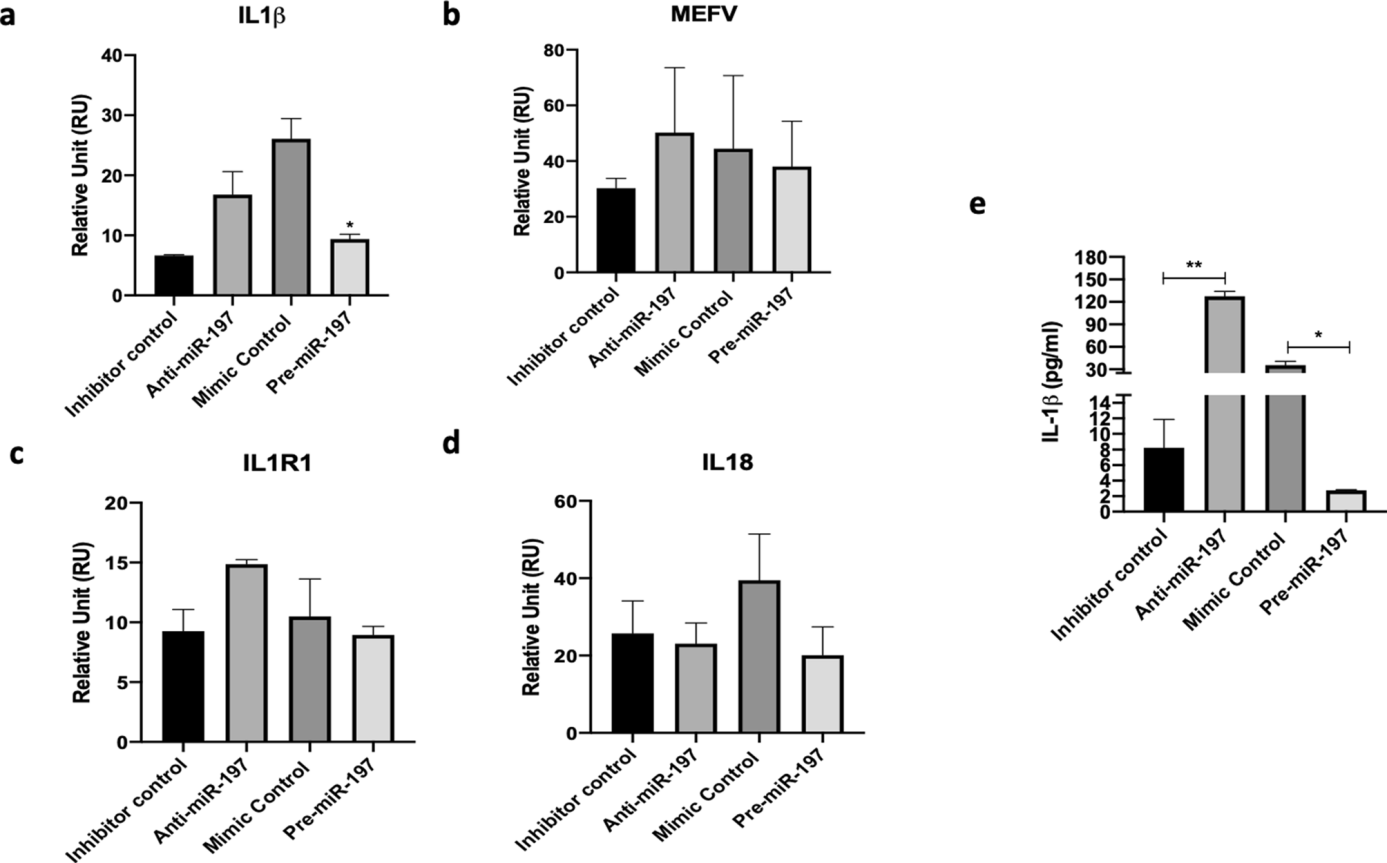
miR-197-3p suppresses the secretion of IL-1β and anti-miR-197 transfection increases the expression of inflammatory cytokines and genes. **(a)** *IL-1β,* (**b)** *MEFV,* (**c)** *IL1R1* and (**d)** *IL-18* gene expression level in the THP-1 derived macrophages cells after anti-miR-197 and pre-miR-197 transfections. (**e)** ELISA results of IL-1β secretion in the THP-1 derived macrophages after anti-miR-197 and pre-miR-197 transfections. Data represent the mean ± standard deviation from three independent experiments. *P < 0.05, Student’s t-test (two-tailed).

*IL-1β* gene expression level in THP-1 derived macrophages was increased after anti-miR-197 transfection and decreased significantly with pre-miR-197 transfection (Fig. 2a). The same pattern was seen in *MEFV* and *IL1R1* genes (Fig. 2b,c). But, *IL-18* gene expression level decreased with anti-miR-197 transfection. This result indicated that IL-18 production may not be related with miR-197-3p, although the gene level was decreased with pre-miR-197 transfection (Fig. 2d).

THP-1 derived macrophages in the presence of LPS showed increase in *IL-1β* , *MEFV,* and *IL-18* gene expression levels and increased at 48 h and 72 h after anti-miR-197 transfection (Supplementary Fig. 1a–c). Only *IL-1β* gene expression level was decreased after 48 h (significantly) and 72 h followed by pre-miR-197 transfection (Supplementary Fig. 1a). There was no difference in *MEFV* gene expression level after pre-miR-197 transfection (Supplementary Fig. 1b). *IL-18* gene expression levels decreased after 48 h of pre-miR-197 transfection, but this was not continued to 72 h where there was an increase in the gene expression level (Supplementary Fig. 1c). These results indicated that *IL-1β* is the most influenced gene by the miR-197-3p transfections in the SW982, THP-1 cells, and THP-1 derived macrophages.

### miR-197-3p suppresses cell migration in SW982 and THP-1 cells

**Figure 3:**
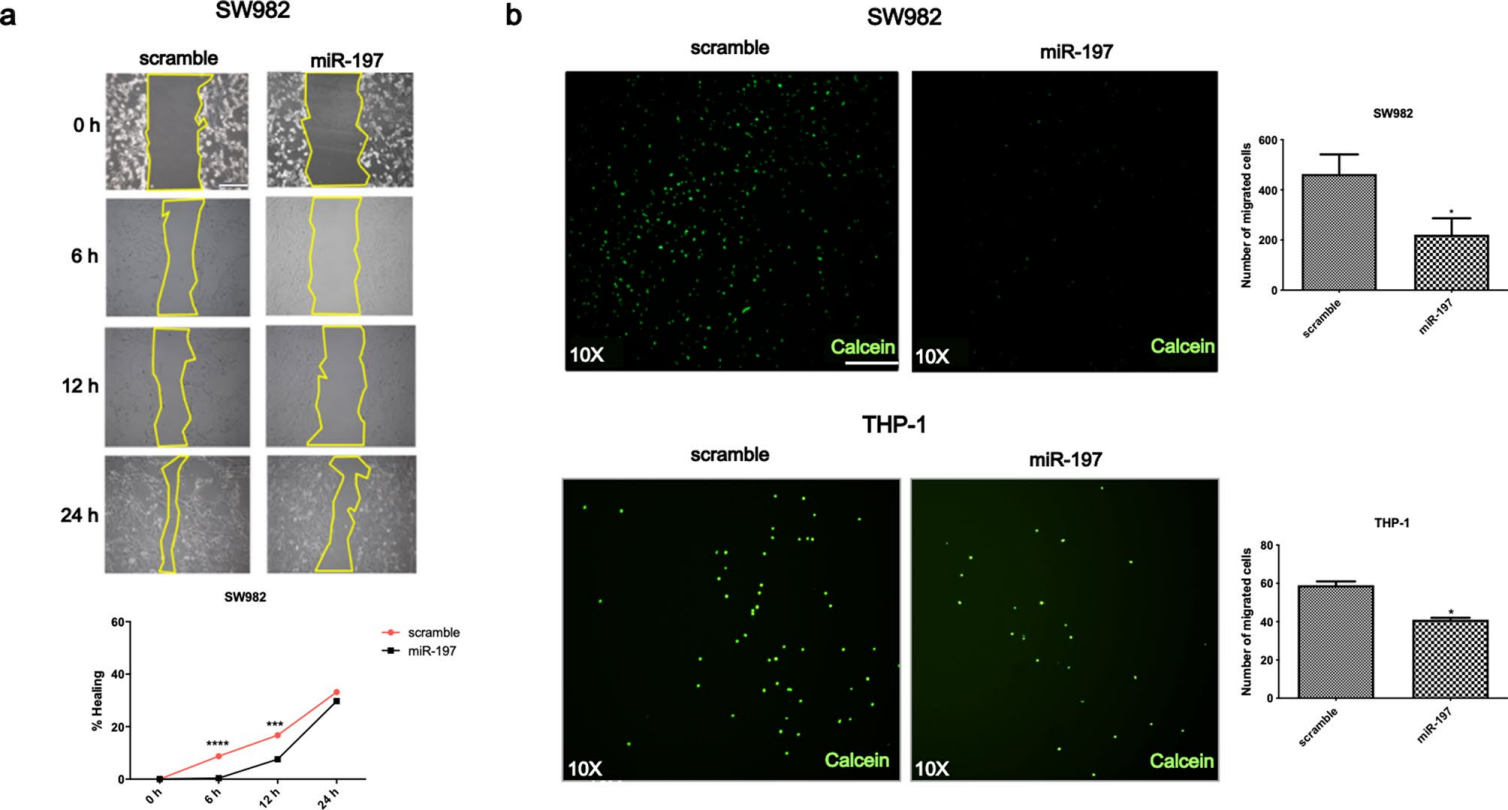
miR-197-3p suppresses the cell migration. **(a)** Wound healing assay of in miR-197-3p or control sequence-transfected (scramble/mimic control) SW982 cell line in time courses (0, 6, 12, 24 h). *** *P* < 0.001; **** *P* < 0.0001, Student’s t-test (two-tailed). (**b)** Filter assay results in miR-197-3p or control sequence-transfected (scramble/mimic control) SW982 and THP-1 cell lines. Calcein (Green): image of living cells passing under the filter. Scale bar indicates 1 mm. Data represent the mean ± standard deviation from three independent experiments. * *P* < 0:05, Student’s t-test (two-tailed).

Both SW982 and THP1 cells, with pre-miR-197 transfection, showed a significant decrease in cell migration at 6 and 12 h, while there was no significant difference with the scramble/mimic controls (Fig. 3a,b). The increase in the expression of pre-miR-197 was accompanied by a decrease in the number of cells migrating under the filter. This decrease in cell migration in pre-miR-197 transfected cells, suggests that this miRNA may influence this pathway or reduce cytokine levels. Thus, downregulation of pro-inflammatory cytokines prevents the activation of signaling pathways related with cell migration in pre-miR-197 transfected cells.

### miR-197-3p suppresses IL-1β secretion

IL-1β secretion was increased significantly following the anti-miR-197 transfection in THP-1 derived macrophages. The opposite effect was obtained in pre-miR-197 transfected cells, where cells showed significant reduction in IL-1β secretion (Fig. 2e). Similar results were observed with the cells triggered with various activators. IL-1β secretion increased at 48 h and 72 h, post anti-miR-197 transfection, in THP-1 derived macrophages after LPS treatment, which was accompanied by a reduction in treated cells (Supplementary Fig. 1d). After anti-miR-197 tranfection, untreated and flagellin treated THP-1 derived macrophages showed increased in IL-1β secretion, yet there was no difference in dsDNA and ATP triggered cells. pre-miR-197 transfected cells in all treatments included untreated cells that secreted less IL-1β (Supplementary Fig. 1e).

### miR-197-3p suppresses caspase-1 activation and apoptosis in SW982 and THP-1 cells

**Figure 4:**
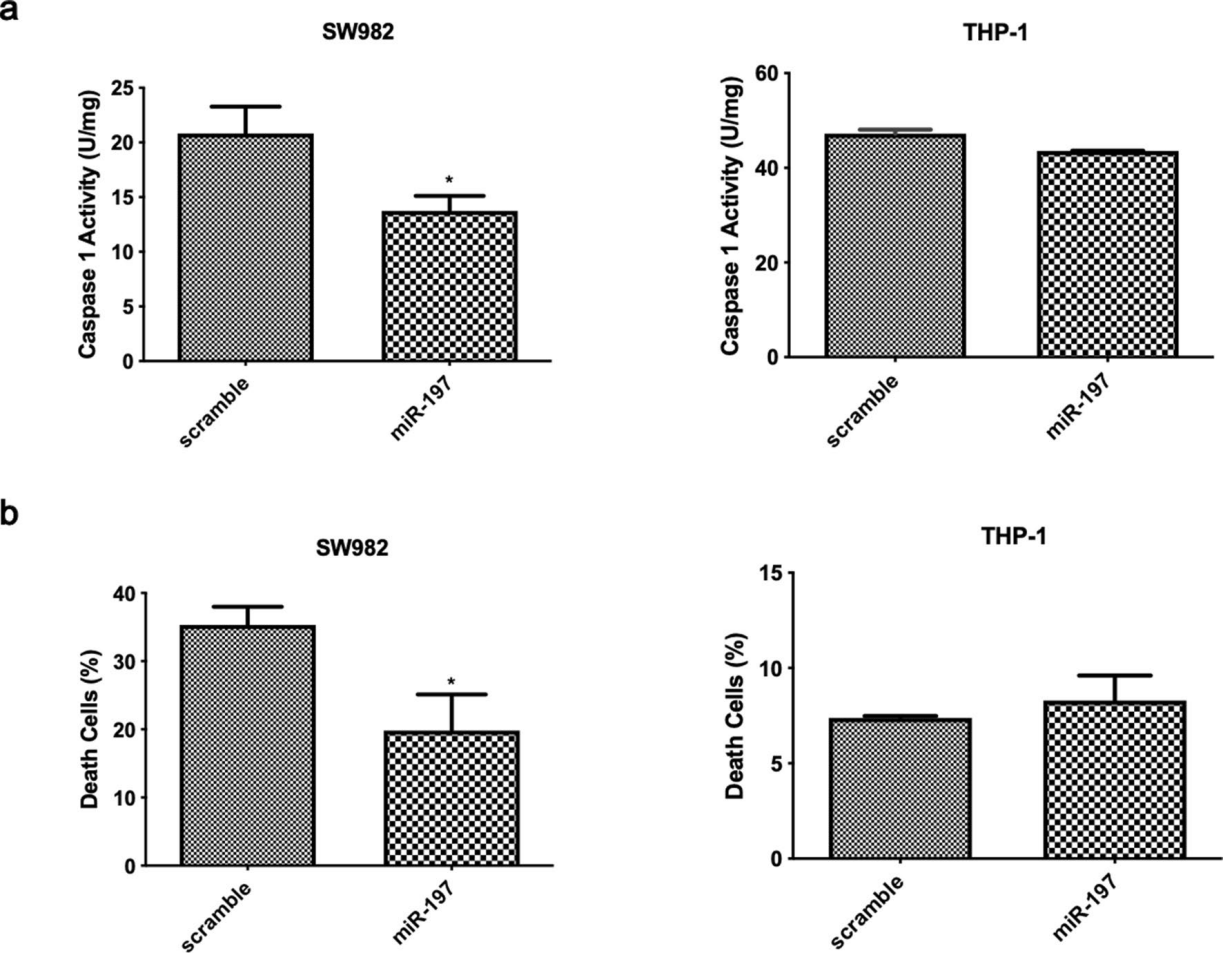
miR-197-3p suppresses caspase-1 activity and apoptosis.** (a)** Caspase-1 activity in miR-197-3p or control sequence-transfected (scramble/mimic control) SW982 and THP-1 cell lines. Data represent the mean ± standard deviation from three independent experiments. * *P* < 0.05, Student’s t-test (two-tailed). (**b)** Apoptosis rate as a death cell in percentage in miR-197-3p or control sequence-transfected (scramble/mimic control) SW982 and THP-1 cell lines. Data represent the mean ± standard deviation from three independent experiments. * *P* < 0.05, Student’s t-test (two-tailed).

In pre-miR-197 transfected SW982 cells, caspase-1 activity was significantly lower, compared to THP-1 cells, where there was non-significant decrease in caspase-1 activity. (Fig. 4a). This decrease in caspase-1 activity in transfected cells supported the concept that this miRNA may have an anti-inflammatory effect. Cells become activated and migrated in response to inflammation signals, to sites of inflammation, where they undergo pyroptosis or apoptosis. Apoptosis was tested as another indicator of cell death shown in inflammation in our study. The cell death rate decreased in pre-miR-197 transfected SW982 cells although in THP-1 cells there was no difference (Fig. 4b).

### miR-197-3p targets IL1R1 in THP-1 cells

**Figure 5:**
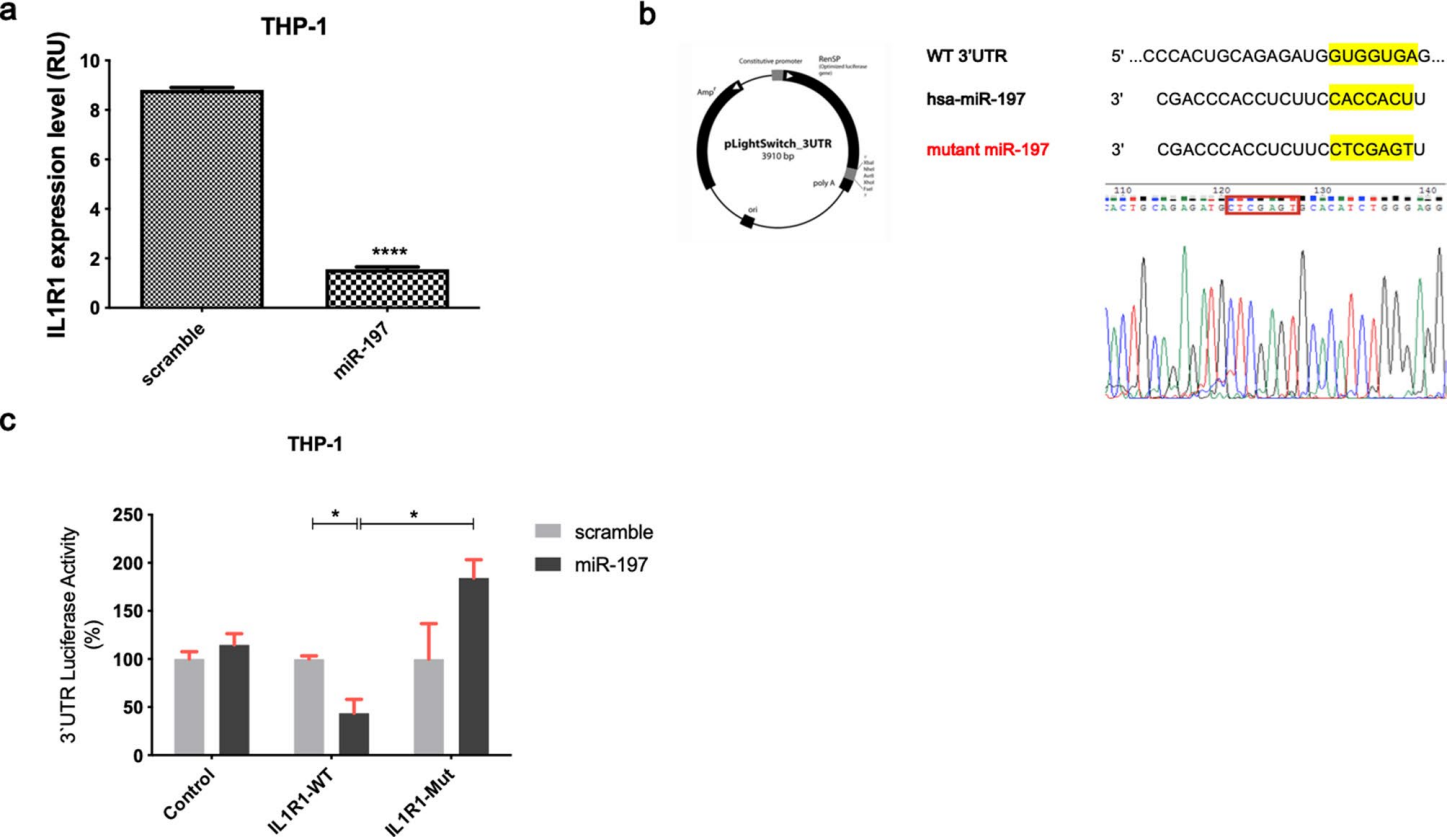
miR-197-3p targets IL1R1 in THP-1 cell line. **(a)** Analysis of IL1R1 expression by qCPR in cells on overexpression of miR-197. Data represent the mean ± standard deviation from three independent experiments. **** *P* < 0.0001, Student’s t-test (two-tailed). (**b)** miR-197-binding site in IL1R1 3′UTR and the sequences of wild-type (WT) and mutant IL1R1 3′UTR in 3′UTR plasmid used in the 3′UTR luciferase experiments are shown. The mutated sequence is indicated by a red frame. Wild sequence: GTGGTGA, Mutant sequence: CTCGAGT. (**c)** WT IL1R1 3′UTR and Mut IL1R1 3′UTR reporter were constructed, and a luciferase reporter assay was performed in THP-1 cells after miR-197-3p transfection. Data represent the mean ± standard deviation from three independent experiments. *P < 0.05, Student’s t-test (two-tailed).

We used the THP-1 cell line for miRNA binding analysis, which naturally expresses pyrin and related proteins. miR-197-3p and its possible target genes were determined by TargetScanHuman (Release 7.2: March 2018). From the given list, *IL1R1* was chosen as a candidate target gene according to score, number of 3P-seq tags and its possible role in inflammation process. We next examined expression of *IL1R1* in pre-miR-197 transfected THP-1 cells. After pre-miR-197 transfection, *IL1R1* gene expression was significantly reduced (Fig. 5a). We sought to analyze a direct interaction of miR-197-3p and *IL1R1* by using 3′UTR luciferase activity assay. This was accomplished with a mutation in the *IL1R1* gene 3′ UTR, which abrogated miRNAs binding to the locus (Fig. 5b). Following validation of mutation; control, wild type and mutant vectors were transfected into THP-1 cells with miRNAs and negative controls. According to the results of 3′UTR luciferase activity assay: miR-197-3p binds to the gene and luciferase signal decreases when there is no mutation in 3′UTR region of *IL1R1* gene. In the case of mutations in this region, the binding region of miRNAs disappears and luciferase signal increases (Fig. 5c). Therefore, miR-197-3p has been shown to directly bind to the 3′UTR region of the *IL1R1* gene experimentally.

## Discussion

Autoinflammatory diseases are caused by dysregulations in the innate immunity, often due to single gene defects^31^. Recent studies have shown that epigenetic control mechanisms, particularly non-coding RNAs, may play a role in the pathogenesis of autoinflammatory and autoimmune diseases. miRNAs, a group of non-coding RNAs have been shown to have activating or suppressing properties in the inflammatory response process. It was found that miRNAs mostly regulate the inflammation process by changing the expression levels of cytokines, transcription factors, and interleukin-related genes^15,16^. In this context, the possible effects of miRNAs on the increased inflammation process in the pathogenesis of FMF were investigated.

Considering the mechanism of inflammation in these diseases, the affected cell quickly regains its homeostasis or goes to pyroptosis by passing through several steps. Alterations in these steps eventually lead to a return to physiological state or a delay or suspension of apoptosis^32,33^. The accumulation of components in the inflammatory region, cell migration, caspase activation and infiltration, together with the activation and release of IL-1β, are important events^34^. The effects of specifically miR-197-3p on the inflammation process in this study were investigated in detail by selecting these steps in functional analysis.

miR-197-3p was found to have anti-inflammatory effect on inflammation related pathways. miR-197-3p has an inhibitory effect on IL-1β secretion, which is preceded by a reduction in *IL-1β* mRNA levels. Therefore, it may play important regulatory role in IL-1β depended inflammation processes. It was determined that pre-miR-197 transfection inhibited cell migration, according to filter experiments in synovial fibroblasts, decreased cell migration according to wound healing experiments at 6 and 12 h and decreased apoptotic rate. Another important process associated with inflammation was the decrease in caspase-1 activity.

Functional studies for miR-197-3p utilized a second cell line model, human monocyte cell line (THP-1), in which pyrin and related proteins are naturally expressed. These cells are known to be involved in the inflammation, and thus a good candidate for gene and functional studies. Cell migration was decreased in pre-miR-197 transfected THP-1 cells. *IL-1β, MEFV, TGF-β,* and *TNF-α* gene expression levels were decreased as well in relation to inflammation. When considered together, miR-197-3p demonstrated an anti-inflammatory effect in monocytes as well and suppressed inflammation. It is recommended to validate miRNAs’ function in at least two different cell lines and determine if similar results are obtained. In this context, similar results obtained in monocytes are very important. For anti-miR-197 transfections, THP-1 derived macrophages were used as a third cell type. Pre-miR-197 transfections were included to the experimental set-ups. Cells shown increased IL-1β secretion after anti-miR-197 transfection as expected. In addition, *IL-1β, MEFV,* and *IL1R1* gene expression levels were also increased after transfection. Similar results were obtained from cells triggered with LPS and other inflammasome activators. These results help to clarify the function of miR-197-3p in inflammation.

Caspase-1 dependent cell death occurs as a result of activation of inflammatory complex is called 'pyroptosis'^35^. Cell death experiments with Annexin V / PI staining measure apoptosis, where caspase-1 activity are correlated to pyroptosis, the mode of death in inflammatory cells. Therefore, caspase-1 activity assays were also evaluated for the determination of pyroptosis. According to the results of caspase-1 activity experiments, miR-197-3p was also found to reduce pyroptosis.

It is possible that miRNAs circulating in exosomes, which are therefore conserved in plasma or serum, can be used as potential biomarkers. In another project in which our group is involved, the presence of candidate miRNAs in exosomes isolated from serum samples of FMF patients were investigated and it was observed that the level of miR-197-3p decreased in exosomes of patients compared to controls (Hacettepe University, Non-interventional Clinical Researches Ethics Board, National ethics committee approval has 13.06.2017 date and GO17/513-11 number. All methods were carried out in accordance with relevant guidelines and regulations. Informed consent was obtained from all subjects or, if subjects are under 18, from a parent and/or legal guardian.) (unpublished data). This data obtained in exosomes supports the hypothesis that the decrease in this anti-inflammatory feature of miRNA systemically in patients leads to increased inflammation and affects the pathogenesis.

Considering the microarray results obtained in our previous study, the downregulation of miR-197-3p in severe FMF patients, further supports the anti-inflammatory effect hypothesis^27^. Therefore, the inflammatory process is triggered, or inflammation may not be suppressed due to decreased expression of miR-197-3p in patients. In our previous study, miR-197-3p has shown a significant decrease in adult FMF patients. In this present study, we analyzed the level of miR-197-3p in pediatric FMF patients. miR-197-3p level was reduced significantly in severe FMF and systemic autoinflammatory diseases (SAIDs) patients, compared to mild FMF patients (Supplementary Fig. 2) in pediatric study group (Hacettepe University, Non-interventional Clinical Researches Ethics Board, National ethics committee approval has 02.12.2015 date and GO15/744-19 number). It is thought that the decrease in this miRNA can be seen in FMF in different age groups, has an epigenetic regulation not age-related and is critical for the inflammation seen in FMF.

The identification of candidate genes of miRNAs is crucial and necessary in order to fully elucidate the functions of miRNAs. Therefore, candidate gene studies for miR-197-3p were performed considering the possible effect on the pathogenesis of FMF. We have shown that the gene target of miR-197-3p was the *IL1R1* gene, both by bioinformatic analysis and 3′UTR luciferase activity assay. A direct linkage was experimentally demonstrated, suggesting that miRNA may be involved in the upstream pathways of inflammation. The *IL1R1* gene encodes a cytokine receptor of the IL-1 receptor family and the encoded proteins serve as IL-1α and IL-1β receptors. It has an important role in immune and inflammatory response regulated by many cytokines^36^. miR-197-3p reduces the number of receptors located on the membrane by suppressing *IL1R1* expression. *IL-1β* decreases NF-κB stimulation mediated by signal transduction and consequently, the precursor *IL-1β (pro- IL-1β*) expression decreases. Given the pathogenesis of FMF, inflammation from mutant pyrin is thus suppressed to some extent. Hypothetically *IL1R1* expression in colchicine-resistant M694V homozygous patients with persistent inflammation may be increased due to decreased miR-197-3p expression. As a result, NF-κB mediated precursor *IL-1β* expression increases, leading to an increase or inability to suppress existing inflammation.

Our results provide insight to the mechanisms underlying autoinflammatory diseases, the epigenetic regulation with miRNAs in terms of these diseases and may contribute to the development of new diagnostic (biomarker) and treatment strategies. Our results revealed the anti-inflammatory effect of miR-197-3p. As miR-197-3p expression was decreased in our patients, this may explain the severe phenotype that was seen in these individuals. In this case, it is predicted that by increasing miR-197-3p expression in patients, an inflammation-suppressing effect can be achieved. Different target genes related with autoinflammation may exist for this miRNA, yet these genes may help us to understand the role of miR-197-3p in inflammation. Although many autoinflammatory diseases are rare, a better understanding of their pathogenesis will help to understand the basic mechanisms of inflammation and innate immunity, which are important for common diseases. By investigating the factors that cause such diseases, new effective and preventive treatment protocols can be developed.

## Methods

### Cell culture and transfection

SW982 and THP-1 cells were obtained from ATCC. THP-1 is a human monocytic cell line derived from an acute monocytic leukemia patient and known as a suitable in vitro cell model to study monocyte and macrophage functions. SW982 cells are human synovial sarcoma cell line and known as fibroblast like synoviocytes. All cell lines were cultured within 3 days in RPMI (Roswell Park Memorial Institute) 1640 (Gibco) medium containing heat inactivated FBS (10% v/v), Penicillin / Streptomycin (1%, 10,000 units/mL of penicillin, 10,000 µg/mL of streptomycin) and L-Glutamine (1%, 200 mM). SW982 cells were transfected with 3 μl of Lipofectamine 2000 (Invitrogen) and 20 nM hsa-miR-197-3p, mirVana miRNA mimic (Ambion) or mirVana miRNA Mimic, Negative Control # 1 (Ambion) according to the manufacturer’s instruction. THP-1 cells were transfected with 1.6 μl of Lipofectamine 2000 (Invitrogen) and 20 nM hsa-miR-197-3p mirVana miRNA mimic (Ambion) or mirVana miRNA Mimic, Negative Control # 1 (Ambion) according to the manufacturer’s instruction. For determining the difference in miR-197-3p expression level, SW982 cell line was treated with TNF-α (10 ng/mL) or TGF-β (5 ng/mL) for 24 h. For anti-miR-197 transfection experiments, 100 nM hsa-miR-197-3p, mirVana miRNA inhibitor (Ambion) or mirVana miRNA Inhibitor, Negative Control # 1 (Ambion) was tranfected to THP-1 cells by using electroporation. After 24 h or 48 h followed by transfection, THP-1 cells were differentiated into macrophages with 200 ng/mL PMA for 24 h. Pre-miR-197 (100 nM) transfections were also repeated with anti-miR-197 transfections to have a same bench experiment set-up. Cells were treated with various inflammasome activators followed by transfection and differentiation. After PMA priming, cells were treated with 1 μg/ml LPS in RPMI 1640 medium for 3 h. For AIM2 or NLRC4 inflammasome activation, 4 μg/ml of dsDNA (40 min) with 4 μl/ml of Lipofectamine 2000 (Invitrogen) or 5 μg/ml flagellin (60 min) with 25 μl/ml DOTAP (Roche), respectively, were mixed in Opti-MEM (Invitrogen). For NLRP3 inflammasome activation, ATP (5 mM) supplemented RPMI 1640 medium was incubated for 30 min. Then, supernatants were collected for immunoblot analysis and cell lysates were used for RNA isolation.

### RNA isolation and quantitative real-time polymerase chain reaction (qRT-PCR)

For expression analysis of related genes; RNA isolation was performed with RNeasy Plus Mini Kit (Qiagen) from pre-miR-197, scramble/mimic control, anti-miR-197, and inhibitor control transfected cells. NanoDrop ND-1000 (Thermo Scientific) spectrophotometer device to measure the concentrations of RNAs isolated from SW982, THP-1 cells, and THP-1 derived macrophages to check RNA quality. The cDNA was synthesized with QuantiTect Reverse Transcription Kit (Qiagen). RNA input concentration of each sample was prepared to be 600 ng/μl. After cDNA synthesis, qRT-PCR reaction was performed for *MEFV, IL-1β, IL-18, TNF-α, TGF-β,* and *IL1R1* genes with iTaq Universal SYBR Green Supermix (BioRad) according to the manufacturer’s guidelines. *GAPDH* gene expression was used as a normalizer. Each sample was run in triple sets and the reaction was carried out on the BioRad IQ5 and Applied Biosystems ViiA 7.

### ELISA

After 24 h or 48 h followed by transfection, THP-1 cells were differentiated into macrophages with 200 ng/mL PMA for 24 h. After PMA priming, cells were treated with LPS, dsDNA, flagellin, and ATP. Then, supernatants were collected for immunoblot analysis. Quantikine ELISA, for human IL-1β/IL-1F2 Immunoassay (R&D Systems) was used according to the manufacturer’s guidelines.

### Apoptosis assay

After transfection of the miRNAs, cell death analysis was performed by flow cytometry using Annexin V-FITC Apoptosis Detection Kit, APOAF (Sigma). Transfected cells were stained with Annexin V FITC Conjugate and/or Propidium Iodide (PI) solution according to the manufacturer’s guidelines and analyzed by BD FACS Aria II device.

### Wound healing assay

SW982 cells were seeded in six-well cell plate at 50% of confluency and transfected with 20 nM pre-miR-197 or scramble/mimic control. After transfection, the cells were expected to reach 90% confluency and then two scratches were made by sterile 10 μl pipette tip. The culture was photographed by Leica IM50 fluorescence inverted microscope at 0, 6, 12 and 24 h.

### Migration assay

Migration assay was performed by the Transwell migration chamber. Polycarbonate membrane filters containing 8 µm pores were placed in 24-well cell culture plate. The filters used in this experimental system have two compartments, namely the upper and lower wells. Firstly, the medium was placed under and above the filter and incubated for 1 h at 37 °C in 5% CO_2_ incubator. Cells transfected with pre-miR-197 or scramble/mimic control for 48 h were harvested and resuspended in serum-free medium. Transfected cells were then transferred to the upper chamber at a density of 3 × 10^4^ cells/well. After 24 h, non-migrated cells were removed by cotton swap. The cells remaining at the bottom of the filter were treated with 1 mM calcein-AM and incubated for 30 min in at 37 °C in 5% CO_2_ incubator. At the end of the incubation, three pictures were taken from the lower area of the filter with the Leica IM50 fluorescence microscope.

### Caspase-1 activity assay

Caspase-1 activity was analysed in pre-miR-197 or scramble/mimic control transfected cells using the Caspase-1 Fluorometric Assay Kit (Abcam) according to the manufacturer’s guidelines. Following the protocol, the samples were measured at Ex/Em = 400/505 nm using SpectraMax M2 microplate reader.

### Mutagenesis of candidate gene 3′UTR vectors

miR-197-3p and its target genes were bioinformatically determined by using TargetScanHuman (Release 7.2). TargetScanHuman database is given a target gene list sorted by cumulative weighted context score and based on 3P-seq tags. 3′ UTR reporter construct for IL1R1 was provided from the LightSwitch 3′UTR Reporter GoClone Collection catalog. The region determined by TargetScanHuman binds miR-197-3p to the candidate gene was amplified by mutagenesis primer. Primers were designed using the QuikChange Primer Design Program (https://www.agilent.com/genomics/qcpd). Mutagenesis PCR was performed using the designed primers with QuikChange II XL Site-Directed Mutagenesis Kit (Agilent Technologies). The reaction was carried out on the Gene Amplification PCR System 9700 (Applied Biosystem) PCR. IL1R1 3′UTR / mut IL1R1 3′UTR reporter constructs were co-transfected into THP-1 cells together with pre-miR-197 miRNA precursor or scramble/mimic control. At 24 h of post-transfection, luciferase activities were analyzed using the LightSwitch Luciferase Assay Reagent (SwitchGear Genomics). These assays are performed by using a pLightSwitch luciferase construct containing the full-length wild-type target gene 3′UTR or its mutant version. This provides functional evidence for direct binding of a miRNA specific to 3′UTR in a cell-based system. The luciferase signal from the cells was measured by Molecular Devices, Spectramax i3x microplate reader.

### Statistical analysis

Differential expression of the genes was normalized according to GAPDH. The results were calculated with relative quantitation (2^−ΔCt^) method by comparison with the control group. After determination of expression level of genes, statistical significance of fold increase/decrease was analyzed using Student’s T-test and Mann–Whitney U test using GraphPad Prism 8.0, Version 8.3.0 (538) software (https://www.graphpad.com/). According to this; p ˂ 0.05 values were considered as significant. All graphics were analyzed and plotted using GraphPad Prism 8.0, Version 8.3.0 (538) software (https://www.graphpad.com/).

## Supplementary Information

Supplementary Figures.
