# Supplementary material for: Familial Mediterranean fever-related miR-197-3p targets IL1R1 gene and modulates inflammation in monocytes and synovial fibroblasts
Source: Sci Rep. 2021 Jan 12;11:685. doi: 10.1038/s41598-020-80097-4 (PMC7803773; doi:10.1038/s41598-020-80097-4)
Supplement: Supplementary file 1 — Supplementary Figures. [file 41598_2020_80097_MOESM1_ESM.docx]

# Familial Mediterranean fever-related miR-197-3p targets *IL1R1* gene and modulates inflammation in monocytes and synovial fibroblasts

Yeliz Z. Akkaya-Ulum PhD^1^, Tayfun Hilmi Akbaba MSc^1^, Zeynep Tavukcuoglu MSc^1^, Jae Jin Chae PhD^2^, Engin Yilmaz PhD^1^, Seza Ozen MD^3^, and Banu Balci-Peynircioglu PhD^1*^

^1^ Department of Medical Biology, Hacettepe University Faculty of Medicine, Ankara,

Turkey

^2^Inflammatory Disease Section, Metabolic, Cardiovascular, and Inflammatory Disease Genomics Branch, National Human Genome Research Institute, NIH, Bethesda, USA

^3^Department of Pediatrics, Division of Rheumatology, Hacettepe University Faculty of

Medicine, Ankara, Turkey


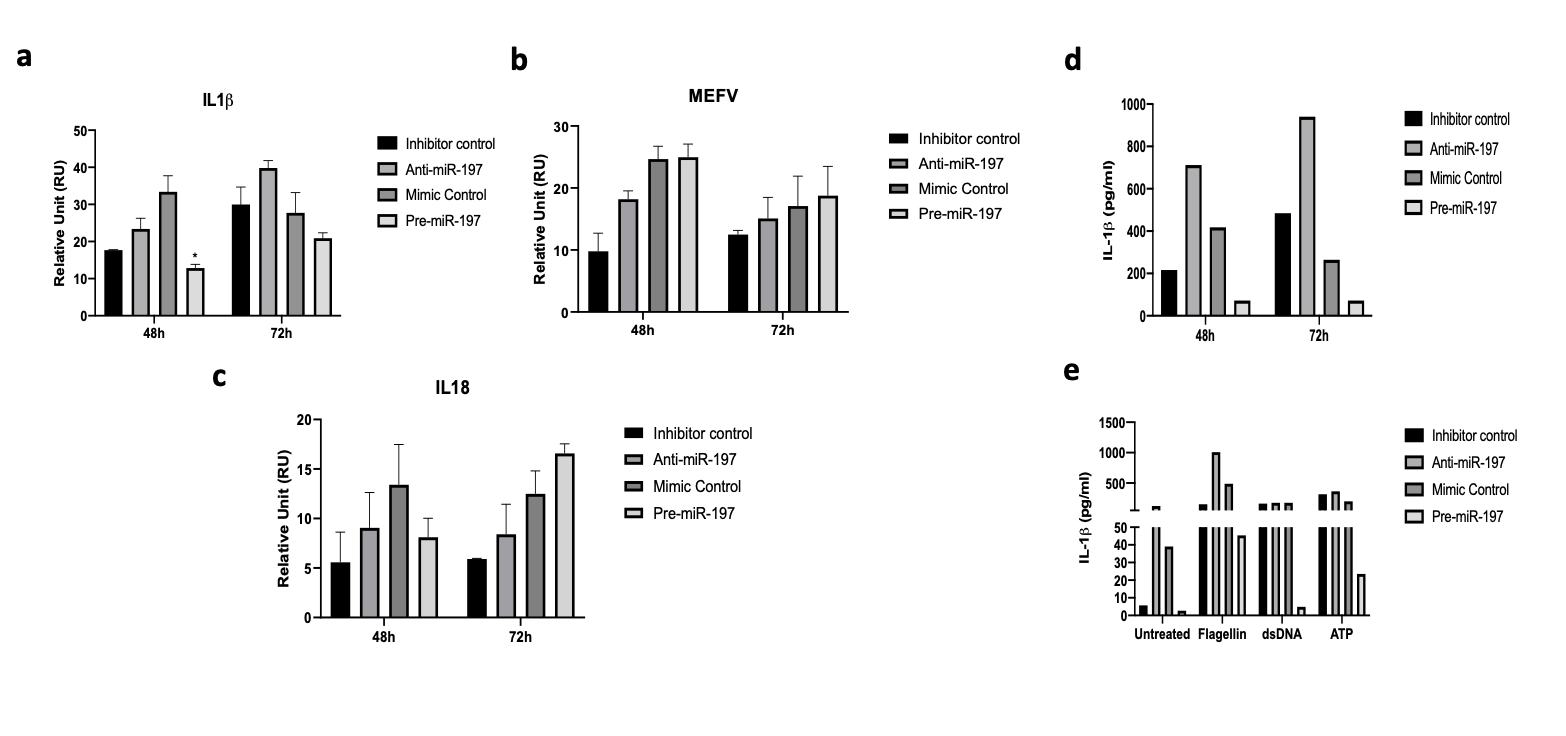


**Supplementary Figure 1.** After anti-miR-197 transfection, cells showed opposite effect of pre-miR-197 transfection. **a)** *IL-1β ,* **b)** *MEFV* and **c)** *IL18* gene expression level in the THP-1 derived macrophages cells treated with LPS after 48h and 72h post-transfection of anti and pre-miR-197. **d)** IL-1β secretion in the THP-1 derived macrophages cells treated with LPS after 48h and 72h post-transfection of anti and pre-miR-197. **e)** IL-1β secretion in the THP-1 derived macrophages cells treated with flagellin, dsDNA and ATP after 48h post-transfection of anti and pre-miR-197. Data represent the mean ± standard deviation from three independent experiments. **P* <0.05, Student’s t-test (two-tailed).


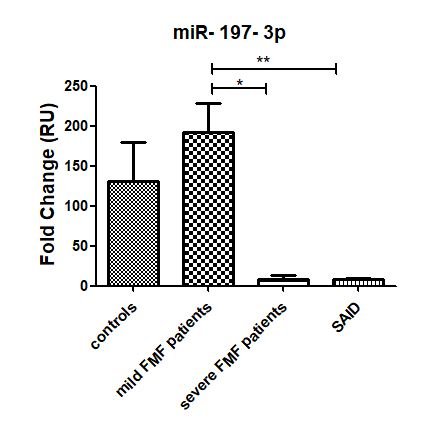


**Supplementary Figure 2. miR-197 expression level was also decreased in pediatric patients.** miR-197 expression level of total blood of pediatric healthy controls, mild FMF patients, severe FMF patients and systemic autoinflammatory diseases (SAIDs) patients by qPCR. ** *P* <0.01, Student’s t-test (two-tailed).
